# Impact of VMAT-IMRT compared to 3D conformal radiotherapy on anal sphincter dose distribution in neoadjuvant chemoradiation of rectal cancer

**DOI:** 10.1186/s13014-018-1187-7

**Published:** 2018-12-03

**Authors:** Hendrik Dapper, Iván Rodríguez, Stefan Münch, Jan C. Peeken, Kai Borm, Stephanie E. Combs, Daniel Habermehl

**Affiliations:** 1 Department of Radiation Oncology, Klinikum rechts der Isar, TU München, Ismaninger Str. 22, 81675 Munich, Germany; 2 Institut für innovative Radiotherapie (iRT), Helmholtz Zentrum München, Ingolstädter Landstr. 1, 85764 Neuherberg, Germany; 3 Deutsches Konsortium für Translationale Krebsforschung (DKTK), Partner Site Munich, Pettenkoferstr. 8a, 80336 Munich, Germany

**Keywords:** Rectal cancer, Anal sphincter, Dosimetric quantification, IMRT vs. 3D-radiation

## Abstract

**Background:**

Neoadjuvant radio- or chemoradiation (nIRT) therapy is the standard treatment for loco-regional advanced rectal cancer patients of the lower or middle third. Currently, intensity modulated radiation therapy (IMRT) is not the recommended radiation technique even though IMRT has advantages compared to 3D-radiation regarding dose sparing to organs at risk like small bowel and urinary bladder. So far, the benefit of IMRT concerning the anal sphincter complex is not examined. With this study we intended to evaluate the dose distribution on the anal sphincters of rectal cancer patients treated with IMRT in comparison with 3D-techniques.

**Methods:**

We selected 16 patients for the IMRT-group and 16 patients for the 3D-group with rectal cancer of the middle third who were treated in our institute. All patients received 45 Gy in a chemoradiation protocol. Patients in both groups were matched regarding stage, primary tumor distance to the anal verge and size of the tumor. We delineated the internal and external anal sphincters, the addition of both sphincters and the levator ani muscle in all patients. Subsequently, we evaluated and compared dose parameters of the different sphincters in both groups and analysed the configuration of the isodoses in the area of the caudal radiation field, respectively.

**Results:**

Most of the relevant dose parameters of the caudal sphincters (Dmean, Dmedian, V10–V40) were significantly reduced in the IMRT-group compared to the 3D-group. Accordingly, the isodoses at the caudal edge of the target volume in the IMRT group demonstrated a steep dose fall. The levator ani muscle always was included into the planned target volumes and received the full dose in both groups.

**Conclusions:**

The modern VMAT-IMRT can significantly reduce the dose to the anal sphincters for rectal cancer patients of the middle third who were treated with conventional chemoradiation therapy.

## Background

Either neoadjuvant short term radiation therapy (nRT, 5 × 5 Gy) or conventional chemoradiation (nCRT) are the standard treatment protocols for patients with locally advanced rectal cancer (UICC-Stage II or III) of the lower and middle third [1]. In the last years, total mesorectal excision (TME) became the standard of treatment and can provide very good local control rates [2, 3]. Even though local control rates were improved by TME alone, nIRT can significantly reduce the number of loco-regional relapses [2, 4]. Next to improved local control sphincter preservation rates can be increased by nCRT especially for tumors of the lower third [2, 5–11].

Due to improved dose sparing of organs at risk (OAR), intensity modulated radiation therapy (IMRT) has become the sole or equivalent standard in tumors of the pelvic which are treated with (neo)adjuvant, definitive or palliative radiation [12, 13]. This technique is more conformal than conventional radiation techniques. Several studies have compared IMRT of rectal cancer to 3D-conformal radiation therapy. IMRT is usually associated with less dose to the rectum, small bowel and the urinary bladder [14–18]. This also translated into better clinical outcome measured by grade 2 but also ≥3 acute gastrointestinal toxicity, genitourinary toxicity and skin side effects [19, 20]. Despite these advantages of IMRT, due to marginal misses in the first experiences of IMRT in rectal cancer, up to now, 3D-RT is still the recommended technical standard of treatment, even though IMRT is acceptable for special cases that do not meet dose constraints with 3D-RT [21].

In the past it was shown that sphincter function worsened by conventional adjuvant or neoadjuvant radiotherapy of rectal cancer [22–27]. With neoadjuvant radiation before surgery, fecal incontinence was almost twice as high compared to surgery alone [26]. Though, unfortunately, studies which compared IMRT to 3D-radiation did not investigate the dose to anal sphincters. Furthermore, in previous published IMRT-studies, sphincter function (fecal incontinence) was not considered as a clinical endpoint [19, 20]. Though, not in rectal cancer, but in prostate cancer, the relationship between dose and sphincter function in IMRT treated patients was performed [28]. Moreover, the correlation of dosimetric parameters of the anal sphincter with sphincter function in patients with locally advanced rectal cancer treated with nCRT was demonstrated [29]. The evidence for standardized constraints is low. The RTOG 0630 phase II trial, dealing with soft tissue sarcoma, required that less than 50% of the anus should receive 30 Gy [30].

So far, the impact of IMRT on nCRT treated rectal cancer patients regarding dose distribution of the anal sphincters has not been investigated. In addition, up to now IMRT has not been the recommended standard technique for treatment. In this study, we evaluated the effect of intensity modulated radiotherapy versus conventional 3D irradiation on the dose distribution of the anal sphincters to estimate the risk of anal incontinence.

## Methods

### Patient selection and radiation techniques

Between 2008 and 2016 a total of 154 rectal cancer patients were treated with neoadjuvant radiation therapy in our institution, 106 with IMRT (either RapidArc (88) or TOMO (28)) and 48 with 3D-RT. To compare IMRT with 3D-RT, we selected the subgroup which might has the greatest possible benefit of sphincter sparing. These are patients with rectal cancer of the middle third (5–12 cm) that were irradiated with a conventional regimen (total dose 45 Gy, single dose 1.8 Gy). In order to make the two groups as homogeneous as possible and thus comparable, we included only such patients in the study who had T3N+ disease.

Patients in the IMRT-group were treated on a Varian Clinac® DHX linear accelerator (Varian Medical Systems, Palo Alto, CA, USA) using IGRT (image-guided radiotherapy) with kilo-voltage cone-beam-CT scans (CBCT). Regularly 2 arcs were used (6 or 15 MV). Some patients received TOMO-therapy with the TomoTherapy Hi-ART-System (6 MV) (Accuray, Sunnyvale, USA). The dose was prescribed to the median of the PTV (ICRU83). Contouring and treatment planning for the intensity modulated radiation therapy was performed using the Eclipse 13.0 planning system (Varian Medical Systems, Palo Alto, CA, USA). Contouring was done on planning CT scans with 3 mm slice thickness. Patients in the 3D-group underwent chemoradiation between 2008 and 2012. All of those patients were treated with ONCOR – Digital Medical Linear Accelerator by Siemens and planned with Oncentra MasterPlan software Version 3.0 SP1, usually 4 fields were used. Daily image guidance was performed using 2D-MV imaging from 0° and 90°. The dose was prescribed to the reference point (ICRU50/62). Dose constraints for organs at risk (OAR) (femoral heads, small bowel, sigmoid colon, genitals and urinary bladder) orientated on Quantitative Analyses of Normal Tissue Effects in the Clinic (QUANTEC) [31]. There were no constraints for the anal sphincter(s) in both groups.

In all patients, the clinical target volume (CTV) definition generally based upon the recommendations of the RTOG Consensus Panel Contouring [32]. In all patients, next to the primary tumor, the whole mesorectum, the peri-rectal-, pre-sacral- and internal iliac nodes were always included into the CTV. As recommended, the visible external iliac nodes were included in four patients in the IMRT group and three patients in the 3D-group. The lower border was at least 2 cm caudal of the gross disease. Further 0.7–1.0 cm were added to reach the PTV margin.

### Contouring and definition of the anal sphincters

For dose-evaluation we defined the volume of the sphincters in two different ways: Most of the studies that analysed dose distribution to the anal sphincters used a unified approach (“sphincter region”) and combined the internal and external anal sphincters as one, mostly filled volume [28, 29, 33]. The cranial border was usually 1 cm below the ampulla recti. The entire anal canal including the anal mucosa/skin was contoured, (CT hyperdensity). For comparison, we defined the internal as well as the external sphincter as “anal sphincter” (AS) in the same way.

**Fig. 1:**
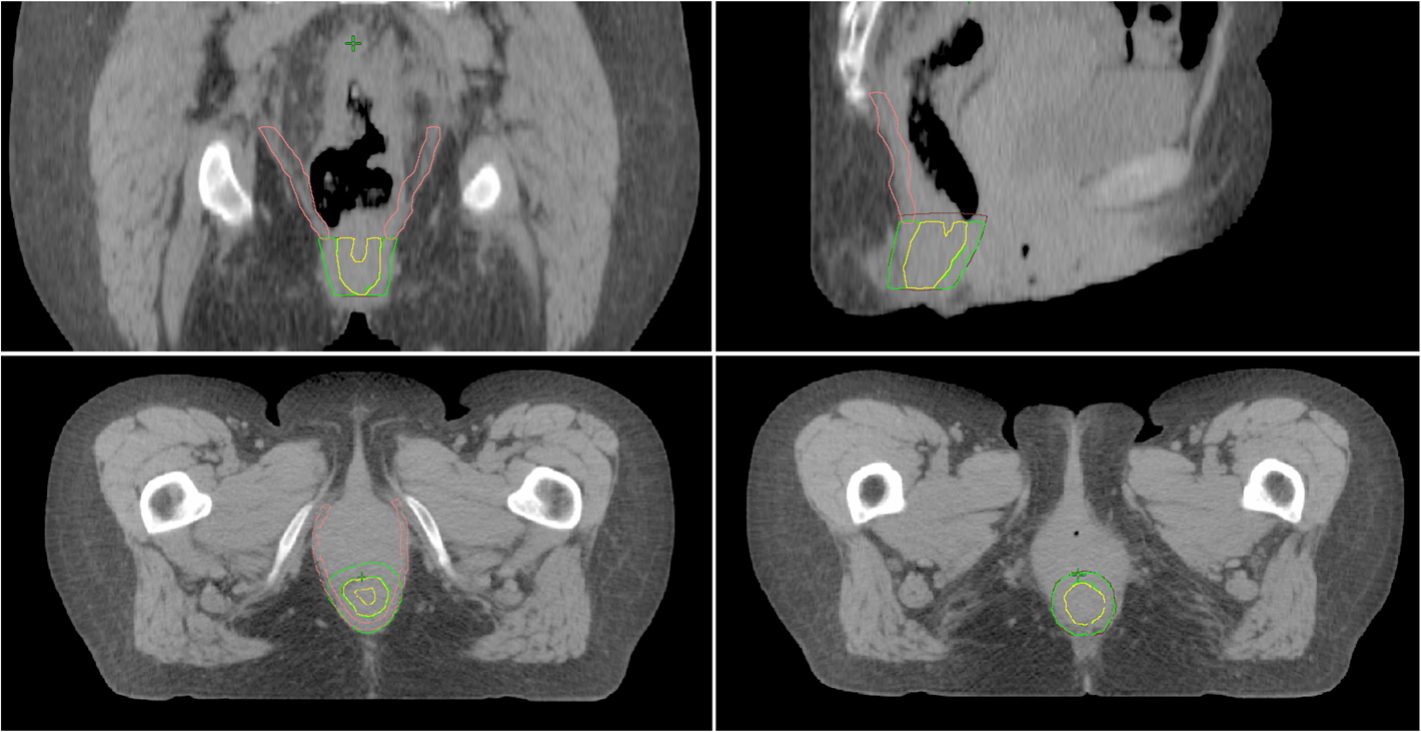
Sphincter delination in a rectal cancer patient. Pink: levator ani muscle, green: external anal sphincter, yellow: internal anal sphincter, brown: anal sphincter (includes internal and external anal sphincter)

As a second, anatomically more detailed approach, we defined the internal anal sphincter (IAS), the external sphincter (EAS), and the levator ani muscle according to the following rules (Fig. 1):

- Internal anal sphincter: Thin muscle which encircles the anal canal. Inner demarcation: Anal mucosa. Outer demarcation: External anal sphincter.
- External anal sphincter: Encircles the internal anal sphincter and merges cranially (at the level of the cranial end of the internal muscle) into the levator ani muscle. Outer demarcation: Central perineal tendon.
- Levator ani muscle: Superior/lateral demarcation: Dorsal part of the pubic body/fascia of the obturator internus muscle. Caudal end: Transition to the external anal sphincter muscle at the level of the cranial internal sphincter muscle.
- Anal sphincter: Includes the internal and external anal sphincters described above.

For the delineation of the sphincters, a matched MRI-scan helped with most individuals, especially for the demarcation of the muscle to the mucosa or skin. For correct comparison, the final contouring was performed on the planning CT-scan in both groups.

### Evaluation of dose and data

We examined the absolute max dose (D98%), absolute min dose (D2%) and the absolute mean and median dose to the entire volume of the different sphincters, respectively. Furthermore, we evaluated and compared the relative dose parameters (V10, V20, V30, V40, V45). In addition, we quantified the differences in dose distribution to the sphincters between the two groups by measuring the longitudinal distance between the 95 and 10% isodoses at the caudal level of the planar centre of the anal canal. For all dose parameters and the longitudinal distance between the isodoses, a Mann-Whitney U test was performed with SPSS 25.0 (SPSS Inc., Chicago, IL, USA) to identify significant differences between the IMRT- and the 3D-group (independent samples). With a *p*-value < 0.5, we assumed that there is a significant difference between the two groups.

## Results

Between 2008 and 2016 a total of 154 rectal cancer patients were treated with neoadjuvant radiation therapy in our institution. Seventy-two (47%) of these patients received a nCRT-concept with a total dose of 45 Gy (single dose 1.8 Gy). About two thirds of this group (49) had a rectal cancer of the middle third. Sixteen patients who fulfilled these criteria were treated with 3D-RT and further 16 patients were matched to this group regarding tumor extension and TNM. This means that finally, a total of 32 rectal cancer patients of the middle third with UICC stadium III (T3N+) treated with long term nCRT between 2008 and 2016 were included and compared in this study. All patients received concomitant chemotherapy, with either 5-Fluoruracil (5FU) on day 1–4 and 29–32 with 1000 mg/qm body surface area (BSA) or Capecitabine 825 mg/qm BSA twice a day, 5 days per week during radiation. The main patients and tumor characteristics are listed in Table 1. The two groups were well balanced regarding tumor extension, distance from the anal verge and volume of the sphincters.

**Table 1:** Patients-, tumor- and sphincter characteristics of rectal cancer patients with T3N+ disease of the middle third

	IMRT	3D
Patients characteristics		
Number of patients	16	16
Median age	61	64
Mean ECOG	1.4	1.3
Sex (Male / female)	10 / 6	11 /5
Tumor characteristics		
G1	1	0
G2	11	12
G3	4	4
Mean shortest distance from anal verge (cm)	5.4	5.5
Mean Tumor extension (cm)	4.6 (3–6)	4.9 (3–7)
Mean sphincter volumes (cc)		
AS	38.4	35.6
IAS	5.4	5.0
EAS	8.6	9.8
Levator Ani	26.1	26.8

*ECOG* Cooperative Oncology Group status, *G* Grading, *AS* anal sphincter, *IAS* internal anal sphincter, *EAS* external anal sphincter

The Dmax (D98%), Dmin (D2%), Dmean and Dmedian of the AS (internal + external muscle + mucosa) were significantly reduced in the IMRT-group compared to the 3D-group (Table 2). The mean Dmax, Dmean and Dmedian in the IMRT group were about 10 Gy lower than in the 3D-group, whereas the mean Dmin was almost the same in both groups (~ 46 Gy). This indicates that at least a small part of the anal sphincter is always covered by the planned target volume (PTV). The dose distribution inside the volume of the anal sphincter shows the effect very clearly (Table 3). From 5 to 30 Gy, the volume of the anal sphincter in the IMRT-group decreased noticeably (99 ➔ 69.4%), whereas the volume changed just marginally from V30 to V45 (76.6 ➔ 73.4%). Furthermore, the V10, V20, V30 and V40 were significantly smaller in the IMRT-group. The dose distribution in the 3D-group changed inversely to the IMRT-group. A large portion of the volume was covered by up to 40 Gy (90.9%) with a break in the maximum high-dose range (45 Gy) (58%), which means that the V45 is significantly lower in the 3D-group than in the IMRT-group (*p* = 0.003).

**Table 2:** Mean absolute dose parameters of different anal sphincters in rectal cancer patients treated with IMRT or 3D-radiation technique

	D98%	D2%	Dmean	Dmedian
Anal Sphincter (Gy)				
IMRT	22,7	45,8	34,3	35,3
3D	31,4	46,2	43,3	45,0
* p*-value	**0,009**	**0,007**	**0,004**	**0,009**
Internal Anal Sphincter (Gy)				
IMRT	23,9	40,2	30,9	29,7
3D	35,3	45,8	43,2	44,5
* p*-value	**0,004**	**0,007**	**0,004**	**0,009**
External Anal Sphincter (Gy)				
IMRT	22,6	40,3	30,5	29,3
3D	30,1	45,5	40,6	42,0
* p*-value	**0,005**	**0,007**	**0,009**	**0,010**
Levator Ani (Gy)				
IMRT	36,6	46,9	44,5	45,9
3D	41,7	46,5	45,5	45,5
* p*-value	**0,000**	**0,005**	**0,008**	**0,005**

*IMRT* Intensity modulated radiation therapy, *3D* 3D-conformal radiation therapy, *Gy* Gray

boldfaced *p*-values are statistically significant

**Table 3:** Mean V5–V45 of different anal sphincters in rectal cancer patients treated with IMRT or 3D-radiation technique

	V5	V10	V20	V30	V40	V45
Anal Sphincter (%)						
IMRT	98,7	91,0	80,1	76,6	74,4	73,4
3D	99,7	98,8	96,5	95,1	90,9	58,0
* p*-value	*0,258*	**0,008**	**0,004**	**0,001**	**0,007**	**0,003**
Internal Anal Sphincter (%)						
IMRT	98,0	87,1	73,6	69,4	66,4	65,3
3D	100,0	100,0	97,5	94,6	88,9	51,2
* p*-value	*0,500*	**0,022**	**0,001**	**0,002**	**0,003**	**0,007**
External Anal Sphincter (%)						
IMRT	98,1	87,2	73,0	68,8	66,0	64,8
3D	99,4	97,3	91,6	88,9	79,4	38,3
* p*-value	*0,258*	**0,010**	**0,004**	**0,005**	**0,007**	**0,001**
Levator Ani (%)						
IMRT	100,0	99,9	98,6	91,7	97,5	97,3
3D	100,0	99,8	99,3	99,3	98,3	65,9
* p*-value	*1,000*	*0,258*	*0,129*	*0,062*	*0,067*	**0,000**

*IMRT* Intensity modulated radiation therapy, *3D* 3D-conformal radiation therapy

boldfaced *p*-values are statistically significant

This pattern of dose distribution was also observed with the internal and external anal sphincter. In the IMRT-group, the volume covered by 10 to 40 Gy was significantly lower, whereas it was significantly higher for 45 Gy.

The situation was different with the levator ani, which is located more cranial than the anal sphincter. It was almost completely covered by the planned target volume in both groups, which has the consequence that the V40 was > 97% in both groups. Only the V45 (prescription dose) was significantly higher in the IMRT-group (97%) compared to the 3D-group (66%). This indicates homogeneous and therefore more adequate dose coverage of the PTV in the IMRT-group.

In summary, except the area where the anal sphincter overlaps with the PTV (prescription dose), the volume of the sphincter (AS, IAS, EAS) can be significantly spared with IMRT. In the IMRT-group, the Dmean, Dmedian and V10–V40 of the caudal anal sphincters were reduced by about one quarter.

**Fig. 2:**
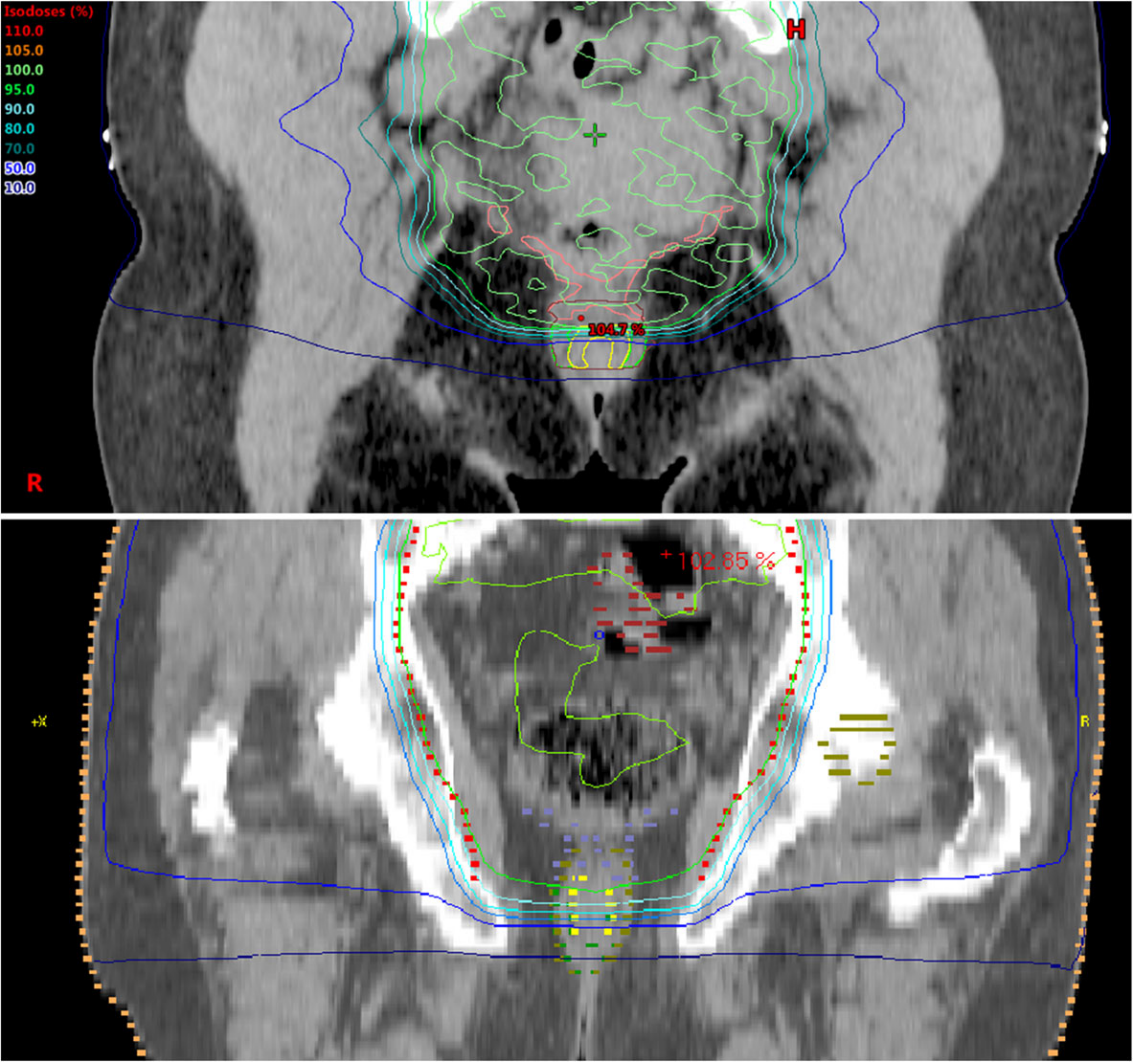
Isodoses in rectal cancer patients treated with neoadjuvant radiation therapy. There is a steep drop of dose from the PTV in caudal extension in the area of the caudal anal sphincters with IMRT (left), whereas there is a much wider distance between the different isodoses in 3D-radiation technique (right)

The detailed evaluation of the isodoses in the area of the caudal PTV and sphincter complex confirmed the results of the dose distribution on the lower anal sphincters. Overall, there was a steep dose gradient in the IMRT-group compared to the 3D radiation-group. Basically in this caudal area of the PTV at the transition to the sphincters, the dose coverage of the PTV was much more homogeneous and better than in the 3D-group. This is a reasonable explanation for significantly higher V45 values in the IMRT- group. Logically, the distance between the different isodoses in the 3D-group was much wider than in the IMRT-group. In the IMRT-group, the longitudinal distance of the 95% isodoses to the 10% isodoses was in mean 1.87 cm (1.59–2.04 cm), while in the 3D group it was 2.4 cm (2.1–2.77 cm) (Fig. 2). This difference was highly significant (*p* = 0.001). The largest distance in the IMRT-group (2.04 cm) was still below the shortest distance in the 3D-group (2.1 cm).

## Discussion

We were able to show that in IMRT-treated rectal cancer patients of the middle third most dose parameters (Dmean, Dmedian, V10–V40) for the lower anal sphincter were significantly reduced using IMRT compared to 3D-technique. The mean Dmean of 30.5 Gy of the EAS in the IMRT-group is equal to the results of Chen et al. for 3D-treated patients (28 Gy), whereas the Dmean in our 3D-group is significantly higher (40.6 Gy) [33]. These differences can be explained by the higher mean tumor location of the patients in the cohort of Chen et al.

### Dose and sphincter function

Long-term functionality of the anal sphincters correlates with the dose to the sphincter complex. Most studies with large patient collectives, in which sphincter dose distribution was correlated with sphincter function, dealt with prostate cancer. The dose prescription in a treatment of prostate cancer is usually much higher (68–78 Gy) than of a long term nCRT concept of the rectum (45 Gy). In an analysis of 414 patients treated with 70 Gy to the prostate or postoperative region, Alsadius et al. could show that long-term fecal leakage largly increases with ≥40 Gy compared to a mean dose < 40 Gy [28]. Buettner et al. quantified dose distribution to the anal-sphincter region and sphincter control of 388 prostate cancer patients treated with 3D-technique [34]. Dose-surface maps were created to estimate the dose of the surface of the anal canal. Furthermore, DVHs and DSHs were determined. Subjective sphincter control correlated significantly with the dose of the sphincters. Optimal cut-points of 47 Gy to the anal sphincter and 45 Gy to the anal surface were found. The relative risk of unsatisfactory subjective sphincter control was also higher for patients receiving ≥30 Gy. One study was found dealing with sphincter function and the correlation of dose in nCRT treated rectal cancer patients. In this study, Arias et al. compared dose distribution and fecal incontinence in rectal cancer patients treated with chemoradiation. Patients with V20 > 0 had significantly more often poor sphincter function (Mean Wexner score of 5.5) compared to those with V20 = 0 (*p* = 0.008). In median the V20 was 34.9% (4.5–85.2%). The authors recommend a sphincter dose constraint of V20 = 0, if possible. In our study, the median V20 was 85.8% in the IMRT-group and 100% in the 3D radiation-group. The lowest V20 in the IMRT-group was 14.9% although we used the same method in contouring of the AS. We assume that the great difference in the V20 is related to the differences in the patient population. Arias et al. selected patients, who received sphincter-preserving surgery and who were more than 2 years free of overall relapse at the time of the study. In our study the majority of the patients had tumors starting from 5 to 6 cm from the anal margin, which resulted in an overlapping of the PTV with the cranial aspect of the AS in most of the cases. Nevertheless, a big difference between the two techniques occurred in V20 of the AS (IMRT: 80.1% vs. 3D: 96.5%; *p* = 0,004). Ultimately, V20, V30 and V40 of the AS, which were found to be relevant in prostate cancer studies, were significantly reduced in the IMRT group compared to 3D-technique.

### Target volume definition and the effect on the anal sphincters

In rectal cancer, a high inter-observer variability in the definition of the clinical target volume in rectal cancer patients exist [35–37]. The caudal border and the ischioanal fossa are two of the structures which usually deviate most from contouring standards [37].

The fact, that loco-regional recurrences of rectal cancer usually occur posteriorly or laterally [38, 39] to the target volume, gives rise to the problem, that in rectal cancers, which are located near to the sphincter complex, an adequate CTV margin is needed. Subsequently, this results in a subtotal coverage of the anal sphincters by the PTV. On the other hand, especially if sphincter preservation is intended, a maximal protection of the sphincters has to be aimed. The common RTOG Consensus Panel recommendations define a caudal extension of the CTV of 2 cm to gross disease with an inclusion of the entire mesorectum to the pelvic floor, even in upper rectal cancers [32]. Roels et al. published recommendations for CTV delineation in advanced rectal cancer based on an analysis of local recurrences and lymph node involvement. They defined pelvic subsites for loco-regional recurrence. The inferior pelvic subsite, which includes the anal sphincter complex, was at risk of locoregional recurrence, when the tumor was within 6 cm from the anal margin or in those patients who were treated with abdominoperineal resection. For the reasons stated above the authors recommend an inclusion of this region in the CTV for patients with a tumor located ≤6 cm from the anal verge or in a situation where the tumor infiltrates the anal sphincter complex and an abdominoperineal resection has been planned. The current consensus guidelines (ESTRO35) differ for this caudal area. They recommend that the ischiorectal fossa should only be included in case of invasion of the external anal sphincter or the ischiorectal fossa [40].

The levator ani muscle is located more cranially to the caudal sphincter complex and is mostly completely covered by the PTV. This leads to a Dmean of about 45 Gy and a V40 of about 98% no matter which of the radiation techniques is used. Because of its cranio-caudal extension along the perirectal fat tissue, this structure is located in a high risk area for loco-regional recurrence. This means, that dose sparing is not desired or useful and more conformal radiation techniques couldn’t improve any protection of this structure.

**Fig. 3:**
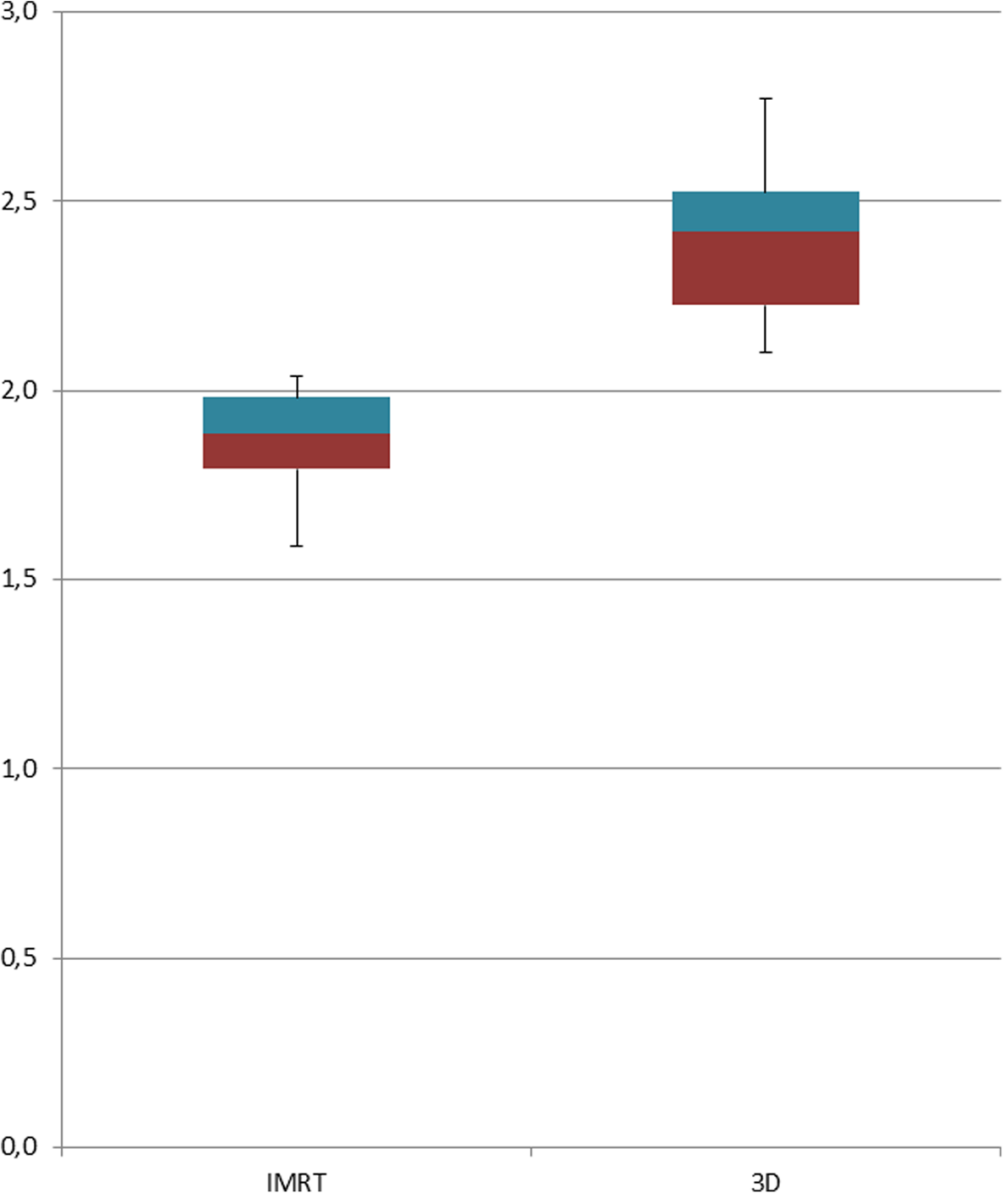
Boxplot of the distance from the 95 to 10% isodose in centimetre for two groups of 16 patients treated with different radiation techniques. The measurement was longitudinal (cranio-caudal) of the planar centre of the anal canal

In our study, the majority of the patients in both groups had cancers starting from 5 to 6 cm above the anal verge. Analogous to the recommendations of Roels et al., in these patients a caudal extension of 2 cm plus about 1 cm PTV margin results either in an overlap or a total coverage of the caudal sphincter muscles with the PTV (Fig. 3).

Regarding dose coverage of anal sphincters, two groups of middle third rectal patients can be identified. In patients, where the primary tumor is in close proximity to the anal sphincter complex, the sphincters should be included into the CTV. This was the case in exactly 8 of 16 patients in each group. The AS was completely covered by the previous PTV, which leads to a V40 of about 100% of the caudal anal sphincters. These patients do not benefit from sphincter protection by IMRT. In patients with a primary tumor starting 1 to 2 cm above the sphincter (also 8 of 16), the sphincter complex only partially overlaps with the PTV. These patients benefit greatly from a steep dose drop of IMRT compared to the 3D-irradiation (Fig. 3). The significant improvement of Dmin, Dmean as well as the V10–V40 in the IMRT-group compared to the 3D-group is supported by the fact that the 8 patients with total anal sphincter inclusion were part of the comparison. As it is known, that pathologic complete response is associated with higher radiation dose and finally improved outcomes, IMRT provides another advantage of local dose escalation (boost) without increased toxicity [41]. In rectal cancer patients of the middle third, this reduced target volume has often some distance to the sphincters and offers another opportunity of sphincter sparing.

### Limitations

The main limitation of this study was the retrospective character. Furthermore, to make a comparison as accurate and meaningful as possible, many of the treated rectal cancer patients could not be included into the study. Unfortunately, this ultimately led to a relatively small number of patients for the final comparison. However, we were able to make the two groups very homogeneous, which ultimately led to a good comparability, in which significant differences in the dose at the sphincters could be shown.

## Conclusion

In patients where the anal sphincter complex does not have to be included into the clinical target volume, IMRT can significantly reduce the mean dose distribution at the anal sphincters for rectal cancer of the middle third that were treated with long-term chemoradiation therapy.

## Abbreviations

3D: 3 dimensional
AS: Anal sphincter
BSA: Body surface area
CBCT: Cone-beam CT
CT: Computed tomography
CTV: Clinical target volume
E. g.: For example
EAS: External anal sphincter
ESTRO: European SocieTy for Radiotherapy and Oncology
Gy: Gray
IAS: Internal anal sphincter
IMRT: Intensity modulated radiotherapy
MRI: Magnetic resonance imaging
nCRT: Neoadjuvant chemoradiation therapy
nRT: Neoadjuvant radiation therapy
OAR: Organs at risk
PET: Positron emission tomography
PTV: Planning target volume
RC: Rectal cancer
RTOG: Radiation Therapy Oncology Group
TME: Total mesorectal excision
UICC: Union Internationale Contre le Cancer
VMAT: Volumetric modulated arc therapy

## References

[1] S3-Leitlinie kolorektales Karzinom (2017). Langversion 2.0 - November 2017 - AWMF-Registernummer: 021/007OL.

[2] Peeters KC, Marijnen CA, Nagtegaal ID (2007). The TME trial after a median follow-up of 6 years: increased local control but no survival benefit in irradiated patients with resectable rectal carcinoma. Ann Surg.

[3] Heald RJ, Moran BJ, Ryall RD, Sexton R, MacFarlane JK (1998). Rectal cancer: the Basingstoke experience of total mesorectal excision, 1978-1997. Arch Surg.

[4] Enríquez-Navascués JM, Borda N, Lizerazu A (2011). Patterns of local recurrence in rectal cancer after a multidisciplinary approach. World J Gastroenterol.

[5] Sauer R, Liersch T, Merkel S (2012). Preoperative versus postoperative chemoradiotherapy for locally advanced rectal cancer: results of the German CAO/ARO/AIO-94 randomized phase III trial after a median follow-up of 11 years. J Clin Oncol.

[6] Cammà C, Giunta M, Fiorica F, Pagliaro L, Craxì A, Cottone M (2000). Preoperative radiotherapy for resectable rectal cancer: a meta-analysis. JAMA.

[7] Sauer R, Becker H, Hohenberger W (2004). Preoperative versus postoperative chemoradiotherapy for rectal cancer. N Engl J Med.

[8] Roh MS, Colangelo LH, O'Connell MJ (2009). Preoperative multimodality therapy improves disease-free survival in patients with carcinoma of the rectum: NSABP R-03. J Clin Oncol.

[9] Gerard J-P, Chapet O, Nemoz C (2004). Improved sphincter preservation in low rectal cancer with high-dose preoperative radiotherapy: the Lyon R96-02 randomized trial. J Clin Oncol.

[10] Chen C-H, Wei P-L, Hsieh M-C (2016). The outcomes of therapeutic decision in lower 3^rd^ rectal cancer patients. Medicine (Baltimore).

[11] Wagman R, Minsky BD, Cohen AM, Guillem JG, Paty PP (1998). Sphincter preservation in rectal cancer with preoperative radiation therapy and coloanal anastomosis: long term follow-up. Int J Radiat Oncol Biol Phys.

[12] Deutsche Krebsgesellschaft, Deutsche Krebshilfe (Leitlinienprogramm Onkologie). Interdisziplinäre Leitlinie der Qualität S3 zur Früherkennung, Diagnose und Therapie der verschiedenen Stadien des Prostatakarzinoms, Langversion 5.0 AWMF); 2018. http://www.leitlinienprogramm-onkologie.de/leitlinien/prostatakarzinom/ [accessed last checked: November 3, 2018].

[13] Engstrom PF, Arnoletti JP, Benson AB (2010). NCCN clinical practice guidelines in oncology. Anal carcinoma J Natl Compr Canc Netw.

[14] X-l D, Tao J, X-g S (2012). Intensity-modulated radiation therapy for advanced cervical cancer: a comparison of dosimetric and clinical outcomes with conventional radiotherapy. Gynecol Oncol.

[15] Viani GA, Viana BS, Martin JEC, Rossi BT, Zuliani G, Stefano EJ (2016). Intensity-modulated radiotherapy reduces toxicity with similar biochemical control compared with 3-dimensional conformal radiotherapy for prostate cancer: a randomized clinical trial. Cancer.

[16] Kwak Y-K, Lee S-W, Kay CS, Park HH (2017). Intensity-modulated radiotherapy reduces gastrointestinal toxicity in pelvic radiation therapy with moderate dose. PLoS One.

[17] Arbea L, Ramos LI, Martínez-Monge R, Moreno M, Aristu J (2010). Intensity-modulated radiation therapy (IMRT) vs. 3D conformal radiotherapy (3DCRT) in locally advanced rectal cancer (LARC): dosimetric comparison and clinical implications. Radiat Oncol.

[18] Yamashita H, Ishihara S, Nozawa H (2017). Comparison of volumetric-modulated arc therapy using simultaneous integrated boosts (SIB-VMAT) of 45 Gy/55 Gy in 25 fractions with conventional radiotherapy in preoperative chemoradiation for rectal cancers: a propensity score case-matched analysis. Radiat Oncol.

[19] Huang C-M, Huang M-Y, Tsai H-L (2017). A retrospective comparison of outcome and toxicity of preoperative image-guided intensity-modulated radiotherapy versus conventional pelvic radiotherapy for locally advanced rectal carcinoma. J Radiat Res.

[20] Simson DK, Mitra S, Ahlawat P (2018). Prospective study of neoadjuvant chemoradiotherapy using intensity-modulated radiotherapy and 5 fluorouracil for locally advanced rectal cancer - toxicities and response assessment. Cancer Manag Res.

[21] Benson AB, Venook AP, Al-Hawary MM (2018). Rectal Cancer, version 2.2018, NCCN clinical practice guidelines in oncology. J Natl Compr Cancer Netw.

[22] Emmertsen KJ, Laurberg S (2008). Bowel dysfunction after treatment for rectal cancer. Acta Oncol.

[23] Lundby L, Jensen VJ, Overgaard J, Laurberg S (1997). Long-term colorectal function after postoperative radiotherapy for colorectal cancer. Lancet.

[24] Pollack J, Holm T, Cedermark B (2006). Late adverse effects of short-course preoperative radiotherapy in rectal cancer. Br J Surg.

[25] Birgisson H, Påhlman L, Gunnarsson U, Glimelius B (2005). Adverse effects of preoperative radiation therapy for rectal cancer: long-term follow-up of the Swedish rectal Cancer trial. J Clin Oncol.

[26] Peeters KCMJ, van de Velde CJH, Leer JWH (2005). Late side effects of short-course preoperative radiotherapy combined with total mesorectal excision for rectal cancer: increased bowel dysfunction in irradiated patients--a Dutch colorectal cancer group study. J Clin Oncol.

[27] Ammann K, Kirchmayr W, Klaus A (2003). Impact of neoadjuvant chemoradiation on anal sphincter function in patients with carcinoma of the midrectum and low rectum. Arch Surg.

[28] Alsadius D, Hedelin M, Lundstedt D, Pettersson N, Wilderäng U, Steineck G (2012). Mean absorbed dose to the anal-sphincter region and fecal leakage among irradiated prostate cancer survivors. Int J Radiat Oncol Biol Phys.

[29] Arias F, Eito C, Asín G (2017). Fecal incontinence and radiation dose on anal sphincter in patients with locally advanced rectal cancer (LARC) treated with preoperative chemoradiotherapy: a retrospective, single-institutional study. Clin Transl Oncol.

[30] Wang D, Zhang Q, Eisenberg BL (2015). Significant reduction of late toxicities in patients with extremity sarcoma treated with image-guided radiation therapy to a reduced target volume: results of radiation therapy oncology group RTOG-0630 trial. J Clin Oncol.

[31] Marks LB, Yorke ED, Jackson A (2010). Use of normal tissue complication probability models in the clinic. Int J Radiat Oncol Biol Phys.

[32] Myerson RJ, Garofalo MC, El Naqa I (2009). Elective clinical target volumes for conformal therapy in anorectal cancer: a radiation therapy oncology group consensus panel contouring atlas. Int J Radiat Oncol Biol Phys.

[33] Chen Y-J, Chen MB, Liu AJ, Sanchez J, Tsai P, Liu A (2014). Dosimetric coverage of the external anal sphincter by 3-dimensional conformal fields in rectal cancer patients receiving neoadjuvant chemoradiation: implications for the concept of sphincter-preserving radiation therapy. Biomed Res Int.

[34] Buettner F, Gulliford SL, Webb S, Sydes MR, Dearnaley DP, Partridge M (2012). The dose-response of the anal sphincter region--an analysis of data from the MRC RT01 trial. Radiother Oncol.

[35] Fuller CD, Nijkamp J, Duppen JC (2011). Prospective randomized double-blind pilot study of site-specific consensus atlas implementation for rectal cancer target volume delineation in the cooperative group setting. Int J Radiat Oncol Biol Phys.

[36] Nijkamp J, de Haas-Kock DF, Beukema JC (2012). Target volume delineation variation in radiotherapy for early stage rectal cancer in the Netherlands. Radiother Oncol.

[37] Joye I, Macq G, Vaes E (2016). Do refined consensus guidelines improve the uniformity of clinical target volume delineation for rectal cancer? Results of a national review project. Radiother Oncol.

[38] Syk E, Torkzad MR, Blomqvist L, Nilsson PJ, Glimelius B (2008). Local recurrence in rectal cancer: anatomic localization and effect on radiation target. Int J Radiat Oncol Biol Phys.

[39] Kusters M, Marijnen CAM, van de Velde CJH (2010). Patterns of local recurrence in rectal cancer; a study of the Dutch TME trial. Eur J Surg Oncol.

[40] Valentini V, Gambacorta MA, Barbaro B (2016). International consensus guidelines on clinical target volume delineation in rectal cancer. Radiother Oncol.

[41] Gunther JR, Chadha AS, Shin US (2017). Preoperative radiation dose escalation for rectal cancer using a concomitant boost strategy improves tumor downstaging without increasing toxicity: a matched-pair analysis. Adv Radiat Oncol.

